# Faking it - II: Countering and preventing counterfeiting of drugs

**DOI:** 10.4103/0019-5545.44899

**Published:** 2009

**Authors:** G. Swaminath

**Affiliations:** Department of Psychiatry, Dr. B. R. Ambedkar Medical College, Kadugondanahalli, Bangalore-560 045, Karnataka, India

## A STITCH IN TIME

Despite being one of the most important tools of the physician in ameliorating illness, the singular lack of attention and importance given to the selection and purity of pharmaceutical brands prescribed is shocking. It is often said that the workman is as good as his skill and his tools. Physicians all over the globe spend immense time and effort in upgrading their skills in diagnosis and therapeutic techniques. However, the unshakable faith in the quality of drugs prescribed often results in the change of diagnosis or the therapeutic molecule in case of non-response rather than of the brand, which might be the real culprit.

The problem of counterfeit medicines, rare in the developed countries, is an everyday reality in India and other developing countries.[1] According to a report, Nimulid (Nimesulide) manufactured by Panacea Biotech had more than 35 counterfeits in the market at one time. Counterfeiters not only copy the trademark, the label design and the trade dress of a well-known branded product, they also copy addresses and pose as licensees.[2]

Counterfeit drugs are a major cause of morbidity, mortality, and loss of public confidence in medicines and health structures. Despite close cooperation between drug companies, governments, or international organizations concerned with trade, health, customs and excise, and counterfeiting, the prevalence of counterfeit drugs appears to be rising.[3] The remedy to this global problem lies in appropriate regulatory processes and rules and strict implementation of these, along with coordination between the players at every level from the policy maker to the regulator to the consumer.

The World Health Organisation (WHO) has now taken the lead in the global effort to combat counterfeit medicines.[1] It has created the first global partnership known as the International Medicinal Products Anti-Counterfeiting Taskforce (IMPACT). IMPACT comprises all 193 WHO Member States on a voluntary basis and includes international organizations, enforcement agencies, national drug regulatory authorities, customs and police organizations, non-governmental organizations, associations representing pharmaceutical manufacturers and wholesalers, health professionals and patients' groups.

These groups have joined to improve coordination and harmonization among countries so that eventually the production, trading and selling of fake medicines will cease. To accomplish this mandate, IMPACT will focus on the following five key areas: i) Legislative and regulatory infrastructure, ii) Regulatory implementation, iii) Enforcement, iv) Technology, and v) Risk communication.[1] It is expected to monitor systems, processes and implementation of regulatory and enforcement areas in each country and will advise on measures to effectively implement them.[1]

The government and the regulatory authorities have a major role in servicing and coordination in all five areas, while doctors and pharmaceutical companies have a role in both technology and risk communication (which has been elaborated in this column). To curb the incidence of counterfeit drugs, the governments need to strengthen their drug regulatory authorities and their powers to enforce drug laws and regulations. The governments should re-work the existing law and ensure that selling spurious drugs is no longer considered a minor offence, now punishable by the state drug controller by suspension or cancellation of license.[4] The Mashelkar committee (1993)[5] recommends strict vigilance, with regular surprise checks at pharmacies and for the cause checks (suspicious checks). The report recognizes the lack of infrastructure and personnel to carry out these checks and recommends drastic upgradation and overhaul of the vigilance structure.[5] Incidentally, there are no accessible testing services for fake drugs anywhere. Paradoxically, the most accessible testing service for fake drugs is the free, anonymous service allowing people to check the authenticity of their illegal ecstasy (MDMA) tablets (www.harmreduction.net).[6]

## DESPERATE REMEDIES

The Mashelkar report specifically notes the absence of mention of spurious drug offences in the Indian Penal Code (IPC) and recommends that the offences be made non-bailable and cognizable, and even recommended the death penalty as the maximum punishment for those dealing in spurious drugs.[5] Vietnam, United Arab Emirates, Oman, Kuwait, Qatar and Bahrain are the only countries in the world which have death penalty for this offence.[7]

There has been a variety of innovative solutions from the pharmaceutical companies to reduce counterfeiting.[8] One such technique is radio-frequency identification (RFID) that uses electronic devices to track and identify items, such as pharmaceutical products, by assigning individual serial numbers to the containers holding each product. Work is on for developing an electronic pedigree (E-Pedigree) system to track drugs from factory to pharmacy, thereby preventing the diversion of drugs or counterfeiting by allowing wholesalers and pharmacists to determine the identity and dosage of individual products.[8] A new technique, Raman spectroscopy, can be used to uncover counterfeit drugs while they are still inside their packaging. A novel low-tech device to test and detect counterfeit drugs, by use of a handheld refractometer,[8] which measures specific gravity of certain dissolved drugs, makes it possible to determine the amount of the active ingredient in a tablet. This device could be used as a first line of defense against counterfeits. These are innovations in the developed countries and IMPACT has the mandate to facilitate transfer such technologies useable in local conditions for use in developing countries such as ours. In addition, the WHO has established a Rapid Alert System (RAS), the world's first web-based system for tracking the activities of drug cheats.[8]

It is, however, in risk communication that the government, regulatory authorities, pharmaceutical companies and physicians have their most important role in alerting key audiences, stakeholders and the general public about counterfeits in communities and across countries. Increased public information is essential for patients, dispensers, and doctors who have a right to know if there are suspect goods on the market. These stakeholders should also contribute to detecting counterfeits by reporting and helping to investigate suspicious cases.[1] However, there is an inherent reluctance of drug companies and governments to publicize counterfeiting to health staff and public, as such a maneuver could cause panic and impact sales. They argue that alarm could lead to people rejecting genuine medicines.[3]

Medical journals as stakeholders in healthcare need to examine their role in risk communication. It is seen that most of the literature on fake drugs derives from local investigative journalism with little scientific public health enquiry relative to the enormous scale of this criminal enterprise.[3] Unfortunately, not many medical journals highlight this problem, nor give space to it. Even journals from India, despite the enormity of the problem, have sidestepped this issue. It is to the credit of the British Medical Journal (BMJ) which has consistently published articles and letters on this issue. It is time journals give space to highlight this important issue and sensitize physicians to be cautious on this issue. This article and its predecessor in this column[9] on the problem of counterfeit drugs is an attempt to address this lacuna.

## SILENT TERRORISM

In a country where nearly half the population is illiterate, poverty is still rampant and where consumer awareness is low, the spread of fake and substandard drugs is a cause for alarm.[10] The task of educating the patient falls on the physician, whom the patient trusts. Physicians should familiarize themselves with the drugs most likely to be counterfeited and their identification.[11] They need to personally check the medicines dispensed to the patient as loose pills, wrong drugs with similar brand names being dispensed, as also expired drugs and those with unsealed or changed packaging.[11] Patients ought to be advised to buy medicines from well reputed chemists and take a receipt, which must carry the batch code and advise destruction of label and container after use.[4] Doctors must develop a high level of suspicion for unexplained worsening in a patient or an unexpected side effect with any medication prescribed. Also, if a patient reports that the drug tastes or looks different, if tablets are chipped or cracked, or if the patient experiences burning at the injection site for an injectable drug, they could be receiving a counterfeit product.[11] There should be a system of reporting these incidents to a regulatory agency that will pursue the issue later.

It is time the medical fraternity takes a stand against dispensing of loose drugs by physicians and pharmacists alike. The loose drugs do not mention the name of the drug, its manufacturer, neither the date of manufacture nor the date of expiry. The manufacturers are also more likely to adhere to quality standards in case the drugs bear their name.[12] The quality of loose drugs is also more prone to deterioration in improper conditions. This step could also reduce the risk of dispensing a wrong drug. The dispensing of labeled drugs would also reduce the doctor-patient asymmetry, as the patients would become aware of their prescriptions. Currently some practitioners give loose drugs to their patients and the patients are unaware of the drugs that they are consuming. There is an argument that strip packing leads to ‘Quality Drugs’ and that the right to ‘Quality Drugs’ an essential part of Right to Life.[12] Except for a few states most state pharmacies which cater to government institutions have been buying drugs in strip packing and this is a good sign.

The maintenance of the drug quality is an essential component of medical care.[12] There is a recommendation to impose death penalty to those who produce or sell counterfeit drugs in India, as to quote a previous central health minister, “Profiting from spurious drugs that might harm or kill innocent people is equivalent to mass murder.”[13] This mass murder is unfortunately perpetuated from within the country, is as much a threat to its citizens as a terrorist attack, and should unite all concerned to counter the scourge.

(Note: Spurious, substandard, fake, and counterfeit drugs have been used interchangeably despite having differences in definition, as the focus is on the hazards to the consumer)
